# Selenium Biofortification of Soybean Seeds Influences Physiological Responses of Seedlings to Osmotic Stress

**DOI:** 10.3390/plants10081498

**Published:** 2021-07-21

**Authors:** Lucija Galić, Marija Špoljarević, Elizabeta Jakovac, Boris Ravnjak, Tihana Teklić, Miroslav Lisjak, Katarina Perić, Franjo Nemet, Zdenko Lončarić

**Affiliations:** 1 Department of Agroecology and Environment Protection, Faculty of Agrobiotechnical Sciences Osijek, Josip Juraj Strossmayer University of Osijek, Vladimira Preloga 1, 31000 Osijek, Croatia; marija.spoljarevic@fazos.hr (M.Š.); elizabeta.jakovac@fazos.hr (E.J.); tteklic@fazos.hr (T.T.); miroslav.lisjak@fazos.hr (M.L.); katarina.peric@fazos.hr (K.P.); franjo.nemet@fazos.hr (F.N.); zloncaric@fazos.hr (Z.L.); 2 Department of Plant Production and Biotechnology, Faculty of Agrobiotechnical Sciences Osijek, Josip Juraj Strossmayer University of Osijek, Vladimira Preloga 1, 31000 Osijek, Croatia; boris.ravnjak@fazos.hr

**Keywords:** biofortification, selenium, physiological response, osmotic stress, water deficit, crop improvement, stress tolerance, climate changes

## Abstract

Climate change poses a serious threat to agricultural production. Water deficit in agricultural soils is one of the consequences of climate change that has a negative impact on crop growth and yield. Selenium (Se) is known to be involved in plant defense against biotic and abiotic stress through metabolic, structural, and physiological activity in higher plants. The aim of this study was to investigate the physiological response of Se-biofortified soybean (*Glycine max* (L.) Merrill) seedlings under osmotic stress. For this research, we used biofortified soybean grain obtained after foliar Se biofortification in 2020. The experiment was conducted in a growth chamber with two cultivars (Lucija and Sonja) grown on filter paper in three replicates. The experiment was carried out with two watering treatments: distilled water (PEG-0) and 2.5% polyethylene glycol 6000 (PEG-2.5) on Se-biofortified seeds (Se) and nonbiofortified seeds (wSe). Contents of lipid peroxidation product (LP), free proline (PRO), total phenolic content (TP), ferric reducing antioxidant power (FRAP), and ascorbic acid (AA) were analyzed in 7-days-old seedlings. Significant differences were detected in the Se content of soybean grains between the two cultivars. A milder reaction to PEG-2.5 was observed in cultivar Lucija in both Se and wSe treatments, which might represent the mitigating effects of Se on osmotic stress in this cultivar. Contrarily, in cultivar Sonja, Se adversely affected all analyzed traits in the PEG-2.5 treatment. Ultimately, Se is a pro-oxidant in Sonja, whereas it represents an anti-oxidant in Lucija. In conclusion, different soybean cultivars show contrasting physiological reactions to both osmotic stress and Se. However, the activation of antioxidant pathways in Sonja can also be interpreted as added value in soybean seedlings as a functional food.

## 1. Introduction

Agricultural land is affected with a varying severity of drought, which has become a worldwide problem. Water deficit, extreme temperatures, and low atmospheric humidity lead to drought, which is one of the most limiting factors for better plant performance and higher agricultural yields [1,2]. Drought is by far the most important environmental stress in agriculture, and it is assumed that by 2050, it will contribute to the salinization of more than 50% of arable land in the world [3]. Generally, abiotic stresses are the greatest restriction for crop production worldwide and account for yield reductions of as much as 50% [4,5]. Levels of soil fertility, moisture supply, and other environmental factors influence seed size and seed weight in all crop species [6]. Soybean (*Glycine max* L.) is an important grain legume with unique chemical composition, making it one of the most valuable agronomic crops [7]. Soybean is one of the most commonly consumed legumes worldwide, with 200 million metric tons produced per year [8] and yields highly affected by water supply [9]. The responses of plants to drought stress are highly complex, involving deleterious and/or adaptive changes [2]. The typical response of plants to low soil fertility and/or chronic osmotic stress is the reduction in quantity of seeds produced rather than in their quality [6].

Drought stress leads to the accumulation of reactive oxygen species (ROS) and increased lipid peroxidation [10]. Oxidative stress caused by a variety of active oxygen species formed under drought stress damages many cellular constituents such as carbohydrates, lipids, nucleic acids, and proteins, which ultimately reduce plant growth, respiration, and photosynthesis [11]. At the cellular level, osmotic stress results in dehydration, which provokes alterations in membrane lipid composition and properties [12]. Lipid peroxidation is a complex process associated with the oxidative deterioration of lipids and the production of various breakdown products [13]. The occurrence of lipid peroxidation in biological membranes causes the impairment of membrane-bound receptors and enzymes, and the increased nonspecific permeability to ions as Ca^2+^ [14]. Low levels of lipid peroxidation during drought conditions are connected to drought tolerance [12]. The ability of higher plants to scavenge the toxic oxygen species appears to be a very important determinant of their tolerance to environmental stresses [15]. Oxidative damage in plants is alleviated by the concerted action of both enzymatic and nonenzymatic antioxidant systems [16]. At the cellular level, drought signals promote the production of stress-protectant metabolites such as proline [17]. Proline plays four major roles during stress, i.e., as a osmotic regulator—osmoprotectant, metal chelator, antioxidative defense molecule, and a signaling molecule [18]. Proline contributes to stabilizing sub-cellular structures (e.g., membranes and proteins), scavenging free radicals, and buffering cellular redox potential under stress conditions [18]. Proline biosynthesis from glutamate (Glu) appears to be the predominant pathway under stress conditions [19]. As proline acts as the molecular chaperon, it is able to maintain the protein integrity and enhance the activities of different enzymes [18]. It has been found that proline acts as a storage compound and source of nitrogen that enhances growth after stress [20]. Another important group of compounds with antioxidant properties found in plants are polyphenols, which neutralize free radicals by increasing the activities and expressions of antioxidant enzymes and inhibiting the activities of ROS-producing enzymes [21]. Phenolic compounds are very reactive in neutralizing free radicals by donating a hydrogen atom or an electron and chelating metal ions in aqueous solutions. Additionally, they have multiple biological properties such as antitumor, antimutagenic, and antibacterial properties, and these activities might be related to their antioxidant activity [22]. Ascorbic acid (AA), also known as vitamin C, is one of the most abundant water-soluble antioxidant compounds present in plant tissues, also serving as an electron donor in numerous reactions [23]. Ascorbate has been shown to play multiple roles in plant growth, such as in cell division, cell wall expansion, and other developmental processes [24]. It is expected that drought stress would trigger an increase in the biosynthesis of a major antioxidant compound such as AA, and plants with increased AA levels might have improved tolerance to such stresses [23]. Drought combined with ascorbic acid improves the plant responses to stress, reducing the production of harmful substances [24]. Ascorbic acid, the reduced form of vitamin C, is an essential component of the human diet, and small amounts can prevent the deficiency disease, scurvy, while the accumulation of high levels of ascorbate in plasma and tissue may protect against oxidative damage and limit inflammation [25].

Se is an essential element in animal cells and in the human body, but its importance for plants is still the subject of research [26]. The toxicity or benefits of Se are highly dependent on the amount of Se applied [27]. Low levels of Se can stimulate the antioxidant machinery in plants, but it acts as a prooxidant at high levels [28]. Some of the positive effects of Se on plants are: promoting plant growth, alleviating UV-induced oxidative damage, improving the recovery of chlorophyll from light stress, increasing the antioxidative capacity of senescing plants, and regulating the water status of plants exposed to drought [29]. Additionally, Se can promote the growth and development of plants and increase the tolerance and antioxidant capacity of plants to environmental stresses [28,30] thus helping to attain higher grain yields [4]. Se has been demonstrated to improve plant growth by strengthening the stress tolerance mechanisms such as antioxidant and secondary metabolite metabolism [31]. Despite its manifold positive effects, Se is not considered to be required by higher plants [32], whereas it is an essential trace element for many organisms, including humans [33]. Globally, the human diet is lacking in Se with especially low intakes in vegetarians [34]. Variations in food Se contents greatly depend on soil Se concentrations, consequently also affecting meat and dairy production [35]. It is of great importance to increase Se in the human diet; therefore, Se biofortification in plants is a method for Se-enriched food production [36]. Agronomic biofortification consists of applying fertilizers of mineral elements lacking in the diet in order to increase their concentrations in crops through soil or foliar application [37,38]. The most efficient method of Se biofortification is foliar application with selenate (SeO_4_^2−^) [38]. Different studies have shown that Se could help in the detoxication of ROS and, thus, the enhancement of plant tolerance to oxidative stress [4], and also improve drought resistance by mitigating oxidative stress [39,40]. Hyper-accumulator species have the ability to accumulate Se in the range between 100 and 1000 mg Se kg^−1^ dw (dry weight) without showing toxicity symptoms in contrast to nonaccumulator species of food crops, grasses, and vegetables that hardly accumulate 100 mg Se kg^−1^ before showing symptoms of toxicity [31]. Toxic levels of Se in plants have not been sufficiently investigated; however, it was shown that tolerance varies according to plant species and genotype [41]. Discovering ways to ameliorate the effects of water deficiency on plants will ease competition for freshwater resources, even as the world’s population grows [17]. It was thus hypothesized that the Se biofortification of soybean could affect the metabolic pathways of bioactive compound synthesis and the antioxidant activity of the seedlings grown from the biofortified soybean seeds.

The aim of this study was to investigate the physiological responses of seedlings of the two Se-biofortified soybean cultivars to osmotic stress induced by PEG treatment.

## 2. Material and Methods

### 2.1. Plant Growth Conditions and Treatments

Seeds of two soybean (*Glycine max* (L.) Merrill) genotypes Sonja and Lucija were obtained from Agricultural institute Osijek, Croatia. Foliar Se biofortification was carried out in growing season 2020 under field conditions with 30 g Se ha^−1^ in the form of sodium selenate (Na_2_SeO_4_) according to the wheat foliar application of 10, 30, 100, or 300 g ha^−1^ [42]. The foliar application of Se was performed on 22nd of July during the R1 growth stage. The soil was eutric cambisol anthropogenic soil under a temperate continental climate. Soybean grains were harvested in September, dried to 13% moisture, and stored. Biofortified grains were inoculated with Apron (Syngenta, Basel Switzerland) seed treatment fungicide and air-dried 24 h before setting up an experiment. Seeds of each genotype were sown between filter paper that had been previously soaked with 55 mL of distilled water (dH_2_O, PEG-0) or 55 mL of 2.5% polyethylene glycol 6000 solution (PEG-2.5). Then, 25 seeds were planted per repetition in single filter paper, folded, and rolled into tubes. Three identical replicates of each treatment were placed in the climate growth chamber at the Faculty of Agrobiotehnical Sciences Osijek, Croatia with the following conditions: 25 °C, 12/12 h day/night regime, and 50% humidity under fluorescent lighting of approximately 200 µmol m^−2^s^−1^ at 20 cm. Soybean seedlings were harvested 7 days after sowing (VE phase) and stored in a −80 °C freezer.

### 2.2. Se Content

The Se concentration in soybean grain was determined after digesting in concentrated ultra-pure HNO_3_ and H_2_O_2_ (3:1 ratio) by stepwise heating up to 250 °C using a Milestone Ultra clave for 1 h and 15 min. Extraction was carried out according to the modified methodology of Matusiewicz et al. [43]. Se concentration was determined using a Perkin Elmer Sciex Elan Inductively Coupled Plasma–Mass Spectrometer (ICP-MS). The standard reference material (SRM) SRM 2709 was used [44].

### 2.3. Bioactive Compounds (Proline, Total Phenolics, Lipid Peroxidation, and Vitamin C)

#### 2.3.1. Proline

The content of free proline (PRO) in the tissue of hypocotyl was determined according to Bates et al. (1973) [45]. The tissue was homogenized in liquid nitrogen and weighed (about 0.2 g) into plastic tubes. Proline was extracted from the tissue with 10 mL of sulfosalicylic acid (3%). The tissue was separated from the supernatant by centrifugation at 3500 rcf at 4 °C for 15 min. To 2 mL of the supernatant, 2 mL of acidic ninhydrin reagent (2.5%) and 2 mL of glacial acetic acid were added. The mixture thus prepared was stirred on a vortex shaker and heated for 1 h in a water bath at 95–98 °C. After heating, the mixture was quenched in ice water and 4 mL of toluene was added to each sample. The samples were stirred for 20 s and left at room temperature until the upper toluene layer with proline separated from the lower, aqueous layer. The standard curve was made using the basic standard of a solution of L-proline with a concentration of 20 µg PRO mL^−1^ in the concentration range of 0–20 µg PRO mL^−1^. The proline concentration in the toluene fraction was determined by measuring the absorbance at 520 nm, and it was calculated from a standard curve with known proline concentrations, which were treated in the same way as the samples were. The final results are expressed as μmol proline g^−1^ fresh matter.

#### 2.3.2. Total Phenolics (TP)

The content of total phenols (TP) in hypocotyls of soybean was determined by the spectrophotometric method with Folin–Ciocalteu reagent according to Singleton and Rossi (1965) [46]. The phenols were extracted with 2.5 mL of ethanol (95%) at −20 °C for 48 h from about 0.1 g of tissue macerated in liquid nitrogen. After extraction, the homogenates were centrifuged at 10,000 rcf at 4 °C for 10 min. To a certain volume of supernatant (depending on the expected values of phenol concentration), about 1.5 mL of distilled water (total volume of supernatant and water is 1.6 mL), 100 µL of Folin–Ciocalteu reagent, and 300 µL of Na_2_CO_3_ (saturated solution) were added. A total of 2 mL of the reaction mixture was stirred on a vortex shaker and incubated in a water bath at 37 °C for 60 min. The phenol content of the incubated and cooled mixture was determined by measuring the absorbance on a spectrophotometer at a wavelength of 765 nm. The phenol concentration was calculated from a standard curve with known gallic acid (GA) concentrations, in the range of 0.05 to 0.5 mg GA mL^−1^. The final phenol content was expressed as mg GA g^−1^ of fresh matter. Samples of the standard solution, as well as the sample solution, were prepared in triplicate.

#### 2.3.3. Lipid Peroxidation

Lipid peroxidation was conducted according to Heath and Packer (1968) [47]. The hypocotyl tissue was comminuted with liquid nitrogen to a fine powder. About 0.2 g of chopped plant tissue was extracted with 1 mL of trichloroacetic acid (0.1%). After centrifugation at 6000 rcf at 4 °C for 5 min, 1 mL of thiobarbituric acid (0.5%) in trichloroacetic acid (20%) was added to 0.5 mL of supernatant. The mixture was heated in a water bath at 95 °C for 30 min and then cooled. After cooling, the supernatant was isolated by centrifugation at 18,000 rcf for 15 min at 4 °C. The absorbance of the sample supernatant was measured spectrophotometrically at wavelengths of 532 and 600 nm. Thiobarbituric acid (0.5%) in trichloroacetic acid (20%) was used as a blank. The concentration of lipid peroxidation products (TBARS) was calculated using the molar extinction coefficient (ε = 155 mM^−1^ cm^−1^) and expressed in thiobarbituric acid equivalents (TBA) in units of nmol TBA g^−1^ fresh matter.

#### 2.3.4. Total Vitamin C Content (Ascorbic Acid)

Vitamin C concentration was determined according to the Roe and Kuether protocol (1943) [48] with some modifications. Solutions of TCA 13.3%—trichloroacetic acid, H_2_SO_4_ 65%—sulfuric acid, DNPH 2%—2,4 dinitrophenylhydrazine (2 g of DNPH, 230 mg of thiourea, 270 g of CuSO_4_, and 100 mL of 5 M of H_2_SO_4_), and ascorbic acid (stock solution)—the basic standard for making calibration curves (0.1 mL^−1^) were used. The soybean tissue was macerated with liquid nitrogen, which was wound in plastic tubes from 15 mL, weighing 0.2 g of crushed tissue. Then, 250 µL of soybean juice was pipetted into 15 mL threaded plastic tubes, and the mass of pipetted juice was weighed. Next, 10 mL of distilled water was added to the leaf mass tubes, and 5 mL of distilled water was added to the tubes where the soybean juice was present. After extraction, the samples were centrifuged for 15 min at 4000 rcf at 4 °C. Subsequently, 150 µL was pipetted in two test tubes (2 mL) for the sample and follow-up. For the purpose of the tubes, 175 µL of distilled water, 100 µL of 13.3% TCA, and only a 75 µL sample of DNPH were added, and the prepared extracts were incubated at 37 °C for 3 h. After incubation of the sample and the next probe, 1000 µL of H_2_SO_4_ was added and DNPH (75 µL) was added in the next probe before that. Standards were prepared in the same way as the sample was, so DNPH was added before incubation. From stock, ascorbic acid solutions were prepared for dilution at concentrations of 0, 25, 50, 75, 100, 125, 150, 175, 200, 250, and 275 g mL^−1^. All samples were vortexed on a Varian Cary 50 UV-Vis spectrophotometer and measured for absorbance at 520 nm in a 1 cm glass cuvette.

### 2.4. Analysis of Antioxidant Activity (FRAP)

The ferric reducing antioxidant power (FRAP) method was determined according to Benzie and Straint (1996) with modifications [49]. Freeze-dried material tissue of soybean hypocotyls was weighed (0.2 g) into a plastic tube, and 10 mL of 96% ethanol was added to the sample, closed and shaken for 20 min on the shaker, and then centrifuged for 15 min at 6000 g under 4 °C. The supernatant was transfused with Pasteur-pipettes, put into a 10 mL volumetric flask, and filled with methanol up to the mark. The contents of the volumetric flask were transferred into the scintillation vessel and frozen immediately. A dissolved pellet with 4 mL of n-hexane was centrifuged, and the supernatant was separated in a 10 mL volumetric flask and frozen. A water bath was set to 37 °C. FRAP reagent containing 200 mL of acetate buffer, 20 mL of TPTZ solution, and 20 mL of FeCl_3_ solution was used. Mixed solutions were placed into the water bath at 37 °C. A spectrophotometer was tuned to a wavelength of 593 nm, and the zero was prepared with 1 mL of FRAP reagent and 100 µL of methanol. The preparation of the standard was as follows: pour 100 µL of standard solution + 1 mL of FRAP solution into the cuvette, mix thoroughly using vortex shaker, and incubate for 4 min at 37 °C in water bath. The dry cuvette was set on the spectrophotometer and measured.

### 2.5. Statistical Analysis

Statistical analysis was conducted with the R programming language (4.0.4 version). Three-way type III analysis of variance (ANOVA) was carried out with main effects cultivar, Se-biofortification, and PEG-2.5 treatment, and all assumed interactions. Differences between treatment means were considered significant at the *p* < 0.05 probability level in Fisher’s LSD test (Table 1). Another one-way ANOVA was carried out for the convenient display of all cultivar-Se-treatment combinations in bar graphs. The differences between means were compared using Fisher’s least significant difference at a *p* = 0.05 probability level.

## 3. Results

### 3.1. Se Content in Soybean Grain and Analysis of Variance (Three-Way ANOVA)

Se biofortification resulted in significant increases by factors of 32.67 and 22.83 in grain Se contents in cultivars Lucija and Sonja, respectively. Significant differences were detected between cultivars (Table 1) after foliar Se biofortification in 2020.

Analysis of variance showed significant effects of the cultivar on all analyzed traits except PRO (Table 2). Main effect Se did not significantly affect any of the analyzed physiological traits, while highly significant effects were observed for main effect PEG-2.5. The interaction between Cultivar and Se showed significant effects on all traits except for TP, while the interaction between Cultivar and PEG-2.5 was significant only for PRO and AA. The interaction between Se and PEG-2.5 significantly affected only LP and TP. The three-way interaction did not significantly affect any of the analyzed traits.

Mean values for main effects PEG-2.5 and Se are shown in Table 3. In alignment with the results of ANOVA (Table 2), significant increases in LP and TP were observed in combination with PEG-2.5 and Se-biofortified cultivars over both assessed cultivars.

### 3.2. Concentration of LP, PRO, TP, FRAP, and AA in Soybean Plant Tissue

**Figure 1:**
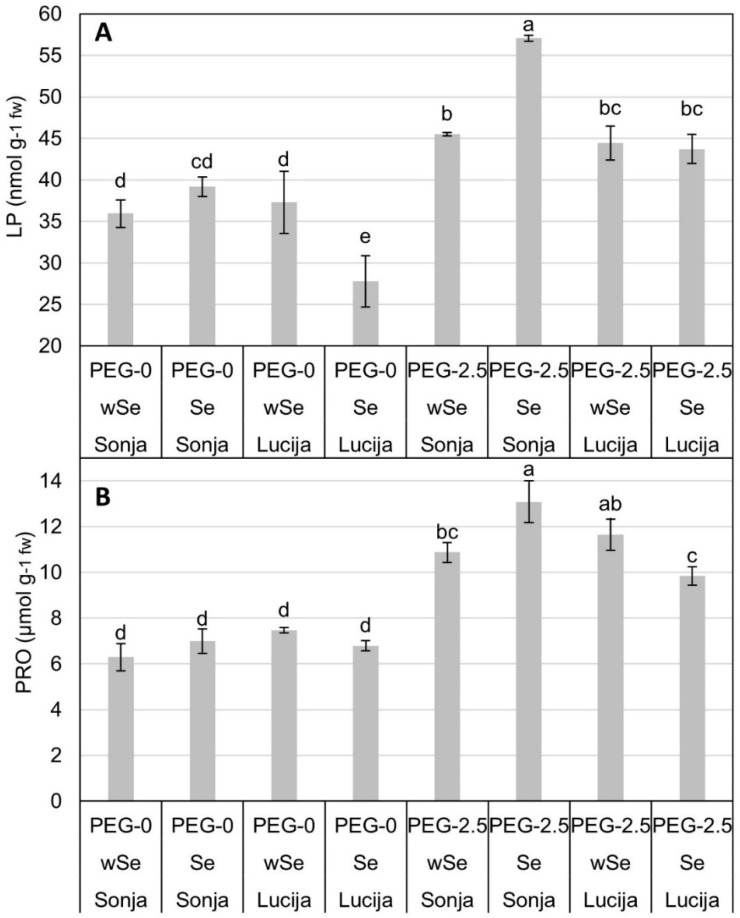
Means and standard errors (SE) of means for PRO (proline) (**A**) and LP (lipid peroxidation product) (**B**). Significance of effects is denoted with different letters at α = 0.05 level.

A 26% increase in LP content (Figure 1A) was observed in plants under PEG-2.5 treatment. Significant differences between LP contents were detected in PEG-0 plants. The significantly highest level of LP in the PEG-0 was in plants with Se in the cultivar Sonja (39.161 nmol g^−1^), while the content of LP did not differ significantly between cultivars Lucija (37.325 nmol g^−1^) and Sonja (35.95 nmol g^−1^) in wSe. In PEG-0, cultivar Lucija showed a significant reaction of LP to Se with the lowest value of 27.769 nmol g^−1^. LP content showed significant differences between groups in PEG-2.5 treatment. The significantly highest value of LP of 57.064 nmol g^−1^ was detected in cultivar Sonja in PEG-2.5 treatment with Se seeds. The content of PRO (Figure 1B) in PEG-2.5 plants was 40.38% higher compared to the PEG-0. There were no significant differences detected between plants in PEG-0. Cultivar Sonja with Se in PEG-2.5 showed the significantly highest level of PRO (13.083 µmol g^−1^). The significantly lowest PRO content in PEG-2.5 with Se was detected in Lucija with 9.84 µmol g^−1^.

**Figure 2:**
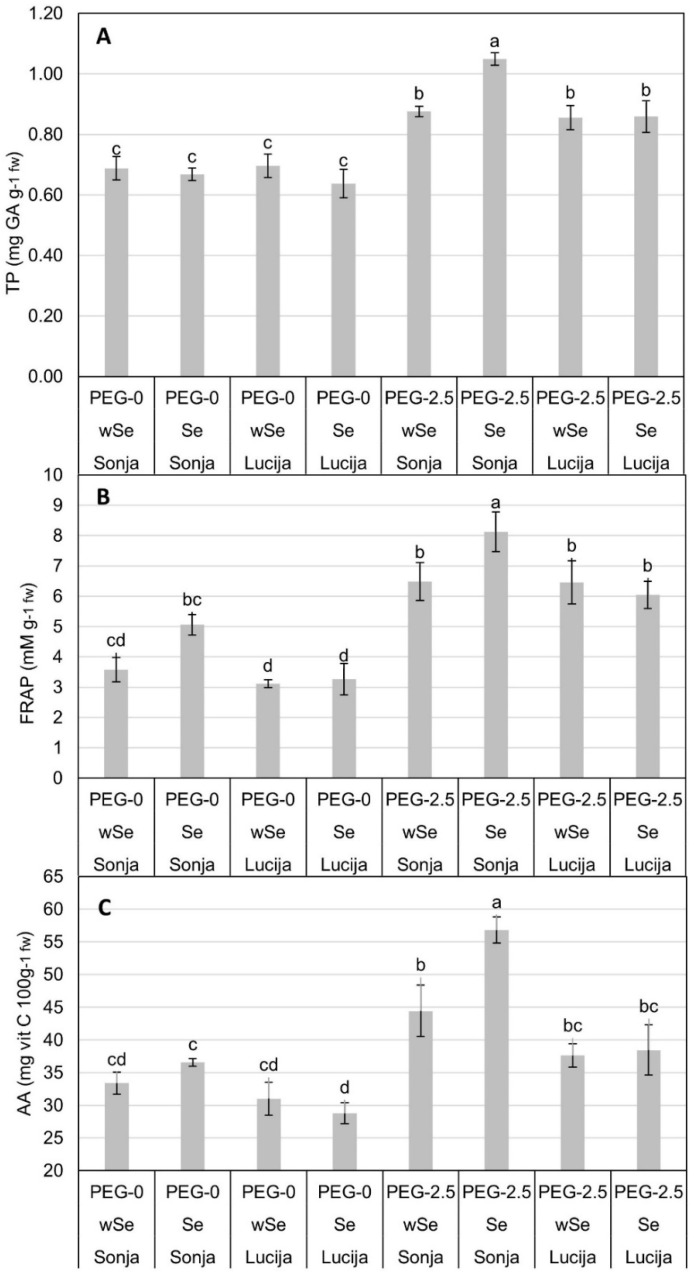
Means and standard errors of mean (SEM) for total phenolic content (**A**), FRAP (total antioxidant activity) (**B**) and AA (ascorbic acid) (**C**). Significance of effects is denoted with different letters at α = 0.05 level.

There were no significant differences detected between groups in PEG-0 plants in TP content (Figure 2A). Plants treated with PEG-2.5 had a higher TP content by 27.17% than PEG-0 plants. The only significant difference in PEG-2.5, compared to other groups, was detected in Sonja with Se with a value of 1.049 mg GA g^−1^ fw, while in the other groups, there were no significant differences. FRAP values (Figure 2B) in PEG-2.5 were higher by 45.29% compared to PEG-0 plants. Significant differences were detected between cultivar-Se groups in PEG-0 treatment. The highest value in the PEG-0 was observed in cultivar Sonja with Se (5.06 Mm g^−1^), while in cultivar Lucija, no significant differences were detected between wSe and Se treatments, although generally, significantly lower values were observed compared to cultivar Sonja. In PEG-2.5 treatment, for FRAP values, a significant difference was found between cultivar Sonja with Se (8.127 Mm g^−1^) and all other groups. Plants in PEG-2.5 showed a higher content of AA by 27.41% compared to plants in PEG-0 (Figure 2C). Significant differences in PEG-0 were detected in Lucija with Se, with the lowest concentration of 28.78 mg AA 100 g^−^^1^, and Sonja with Se (56.794 mg AA 100 g^−1^). In PEG-2.5, Sonja with Se showed the highest and significant value of 56.794 mg AA 100 g^−1^. There were no significant differences detected between the other groups in PEG-2.5.

## 4. Discussion

Significant differences between treatments and cultivars showed contrasting responses of soybean seedlings to Se biofortification in PEG-2.5 treatment (Figure 1A). Similar to our results, PEG-2.5 treatment induced mild stress in soybeans in the study of Basal et al. (2020) [50]. Drought or heat stress can result in the increase in lipid peroxidation [51], while the use of Se at lower doses can stimulate the antioxidant capacity in cucumber (*Cucumis sativus* L.) and reduce lipid peroxidation [26]. In our study, PEG-0 plants with Se in Lucija showed a lower LP by 29.09% compared to Sonja with Se in the same treatment, while LP in plants without Se yielded no significant differences. Our results in Lucija are in accordance with the results in white clover (*Trifolium repens* L.) [39], lettuce (*Lactuca sativa* L.) [52], and in ryegrass (*Lolium perenne* L.) [53] where Se reduced LP. The decrease in LP by exogenous Se may be attributed to its beneficial effects on the antioxidant potential of plants [30]. However, the increase in LP in Sonja with Se (PEG-2.5) was caused by different physiological responses compared to Lucija, which is corroborated by the results of a study conducted on ryegrass where higher doses of Se enhanced the accumulation of the products of LP [53]. Accordingly, Jozwiak and Politycka (2019) [26] conducted research confirming that higher doses of Se can increase LP; therefore, Se at lower concentrations is an antioxidant [54], while high doses can have deleterious effects [53].

Cultivar Sonja in PEG-2.5 treatment with Se showed a 24.79% increase in PRO in seedling tissue compared to Lucija in the same treatment (Figure 1B). Osmolytes, such as proline and glycinebetaine, accumulate under water deficit to help conserve tissue water and protect proteins and cellular membranes from osmotic and oxidative stresses [55]. Proline accumulation is generally regarded as a functional adaption against osmotic stress [56]. The increase in proline accumulation with the increase in severity and duration of drought helps plants maintain tissue water status and avoid drought-induced damage [55]. The increase in TP was detected in cultivar Sonja in PEG-2.5 with Se (Figure 2A). The increase in TP under stress conditions is related to the genetics and growth environment of plants [57]. The antioxidant activity of phenolics is mainly caused by their redox properties, which allow them to act as reducing agents, hydrogen donators, and singlet oxygen quenchers along with their metal chelation potential [58]. Pannico et al. (2020) showed that phenolic compounds increase with Se biofortification in coriander (*Coriandrum sativum* L.), basil (*Ocimum basilicum* L.), and tatsoi (*Brassica rapa* var. *rosularis*) [59]. Phenols have the ability to increase the antioxidant capacity and improve the ability of plants to alleviate oxidative stress [27,60]. Cultivar Sonja showed the highest level of FRAP across both PEG-2.5 and PEG-0 (Figure 2B). Previous investigations have also shown an elevation in the antioxidant activity in Se-treated plants under different abiotic stresses [40]. In a study by Puccinelli et al. (2020), the highest content of antioxidant capacity, total phenol, and rosmarinic acid contents were detected in the Se-treated plants. This could be related to the reaction of plants against the potentially toxic effects of Se in basil [60]. Cultivar Sonja with Se showed the highest content of AA in PEG-2.5 treatment (Figure 2C). AA is an organic acid; under drought stress, respiration is increased and, therefore, these acids act as substrates in the respiration phenomenon [57]. Increasing the AA content in plants can have a triple-positive effect: producing food with a high content of AA for healthy human diets, increasing the postharvest shelf life of products, and increasing the resistance of plants to various kinds of stress [61]. In PEG-0 treatment, decreases in physiological parameters were observed in Se-biofortified plants in cultivar Lucija, while a mild mitigating effect on the osmotic stress in PEG-2.5 treatment with Se was observed through the reduction in proline content. Accordingly, we suggest that biofortified cultivar Lucija might be further examined in field experiments because of its suitability for field crop production. On the other hand, a potentially negative physiological response to Se was observed in cultivar Sonja, where it could act as a prooxidant, presenting a good starting point for the further research of cultivar Sonja for the production of functional Se-enriched food with a high content of phytoactive components.

## 5. Conclusions

Climate change causes shifts in worldwide climatological scenarios with spring droughts becoming more and more frequent [62]. Accordingly, from our results, it can be seen that the Se biofortification of soybean seeds could help mitigate the effect of water deficit in Lucija. High concentrations of LP, PRO, TP, FRAP, and AA suggest that Sonja with Se had a stronger defense mechanism than Lucija in both PEG-0 and PEG-2.5 treatments. Our results can be interpreted in several ways: that Sonja was more sensitive to Se and had a stronger physiological response, or defense reactions in Sonja are slower and were not captured within the growing setup of our study. There is a possibility that a small difference in concentration is sufficient to cause toxicity in Sonja. Our results also suggest that it might be necessary to determine which Se concentrations are optimal for a particular cultivar when it comes to mitigation of the stress effect caused by water deficit. It is necessary to find an appropriate measure for crop improvement to mitigate the negative effects of climate change, and Se biofortification is certainly one of them [11,39,63,64]. From the conducted research, we conclude that Se biofortification in Lucija might be more appropriate for crop improvement, which should be further tested in field experiments. Sonja should be further investigated in light of functional food production in the form of soybean sprouts because Se directly affected the higher production of TP and FRAP and doubled the concentration of vitamin C (AA). Undoubtedly, both cultivars significantly and considerably increased grain Se, which is of great importance for human health.

## Figures and Tables

**Table 1:** Mean values ± standard deviations of Se contents in soybean grains after foliar biofortification with 30 g Se ha^−1^ in 2020. Different letters represent significance of differences at α = 0.05 level.

Treatment	Cultivar	µg Se g^−1^
wSe	Lucija	64.02 ± 36.04 b
	Sonja	101.42 ± 65.87 b
Se	Lucija	2091.67 ± 97.29 a
	Sonja	2315.33 ± 331.8 a
**LSD 0.05**	333.09	

**Table 2:** Results of ANOVA for lipid peroxidation product (LP), proline content (PRO), total phenols (TP), total antioxidant activity (FRAP), and ascorbic acid (AA). Significance of effects is denoted with * (α = 0.05), ** (α = 0.01), and *** (α = 0.001), while *p*-values are given for factors lacking significance.

	LP	PRO	TP	FRAP	AA
Cultivar	**	0.345	*	**	***
Se	0.485	0.783	0.356	0.064	0.061
PEG-2.5	***	***	***	***	***
Cultivar * Se	**	**	0.061	*	*
Cultivar * PEG-2.5	0.492	*	0.086	0.923	*
Se * PEG-2.5	*	0.813	*	0.788	0.103
Cultivar * Se * PEG-2.5	0.937	0.103	0.225	0.625	0.399

**Table 3:** Mean values ± standard error of mean for lipid peroxidation product (LP), proline content (PRO), total phenolic content (TP), total antioxidant activity (FRAP), and ascorbic acid (AA) over main effects PEG and Se. Different letters represent significance of differences at α = 0.05 level.

PEG	Se	LP	PRO	TP	FRAP	AA
PEG-0	wSe	36.64 ± 1.86 b	6.88 ± 0.38 b	0.69 ± 0.02 b	3.35 ± 0.22 b	32.19 ± 1.45 c
	Se	33.47 ± 2.95 b	6.89 ± 0.26 b	0.65 ± 0.02 b	4.16 ± 0.49 b	32.69 ± 1.91 bc
PEG-2.5	wSe	44.97 ± 0.94 a	11.26 ± 0.4 a	0.87 ± 0.02 a	6.47 ± 0.42 a	41.04 ± 2.46 ab
	Se	50.39 ± 3.09 a	11.46 ± 0.85 a	0.95 ± 0.05 a	7.08 ± 0.59 a	47.62 ± 4.54 a
LSD 0.05	-	7.01	1.54	0.094	1.32	8.39

## Data Availability

All data used to conduct this study are available from the corresponding author upon request.
